# Scientific Publications in Nephrology and Urology Journals from Chinese Authors in East Asia: A 10-Year Survey of the Literature

**DOI:** 10.1371/journal.pone.0014781

**Published:** 2011-04-08

**Authors:** Jing Xu, Zhi-Guo Mao, Mei Kong, Liang-Hao Hu, Chao-Yang Ye, Cheng-Gang Xu, Shu Rong, Li-Jun Sun, Jun Wu, Bing Dai, Dong-Ping Chen, Yu-Xian Zhu, Yi-Xiang Zhang, Yu-Qiang Zhang, Xue-Zhi Zhao, Chang-Lin Mei

**Affiliations:** 1 Department of Nephrology, Changzheng Hospital, Second Military Medical University, Shanghai, China; 2 Clinical Laboratory, Fifth Affiliated Hospital of Sun Yat-sen University, Zhuhai, Guangdong, China; 3 Department of Internal Medicine, Changhai Hospital, Second Military Medical University, Shanghai, China; University of Pittsburgh Medical Center, United States of America

## Abstract

**Background:**

Diseases of the kidneys and genitourinary tract are common health problems that affect people of all ages and demographic backgrounds. In this study, we compared the quantity and quality of nephrological and urological articles published in international journals from the three major regions of China: the mainland (ML), Hong Kong (HK), and Taiwan (TW).

**Methods:**

Nephrological and urological articles originating from ML, TW, and HK that were published in 61 journals from 1999–2008 were retrieved from the PubMed database. We recorded the numbers of total articles, clinical trials, randomized controlled trials, case reports, impact factors (IF), citations, and articles published in the leading general-medicine journals. We used these data to compare the quantity and quality of publication output from the three regions.

**Results:**

The total number of articles increased significantly from 1999 to 2008 in the three regions. The number of articles from ML has exceeded that from HK since 2004, and surpassed that from TW in 2008. Publications from TW had the highest accumulated IF, total citations of articles, and the most articles published in leading general-medicine journals. However, HK publications had the highest average IF. Although ML produced the largest quantity of articles, it exhibited the lowest quality among the three regions.

**Conclusion:**

The number of nephrological and urological publications originating from the three major regions of China increased significantly from 1999 to 2008. The annual number of publications by ML researchers exceeded those from TW and HK. However, the quality of articles from TW and HK was higher than that from ML.

## Introduction

Diseases of the kidneys and genitourinary tract are common health problems that affect people of all ages and demographic backgrounds. The increasing incidence and prevalence of chronic kidney disease (CKD) have become a global public health challenge [1]. The screening of 6311 adult (>20 years old) residents of southern China found albuminuria, hematuria, and reduced estimated glomerular filtration rates (eGFR) in 6.6%, 3.8%, and 3.2% of participants, respectively [2]. Chronic glomerulonephritis, usually caused by primary glomerular disease, constituted the leading cause of end-stage renal failure (ESRD) in China, followed by diabetic and hypertensive nephropathies [3]. The annual rate of dialysis in Mainland China was 79.1 per million people (pmp) in 2008 [4]. Renal replacement treatment for patients with ESRD, including maintenance dialysis and transplantation, has posed large economic burdens on patients and the government. Although awareness of treatment options for many nephrological and urological diseases has recently improved, most patients undergoing prostate biopsies in China have exhibited urinary symptoms and elevated prostate-specific antigen (PSA) levels [5], [6]. A cohort study of a Chinese population found a high rate (46.3–61%) of regional lymph node involvement or distant metastasis upon initial prostate cancer diagnosis [6]. However, methods that increase the early detection and downstaging of prostate cancer, such as PSA screening and digital rectal examinations, are not routinely used.

An examination of articles published in the last 10 years (January 1, 1998–August 31, 2008) in all fields of the Essential Science Indicators database ranked China's publication output fifth in the world [7]; however, scientific publications by Chinese authors in the fields of nephrology and urology were not reported. In this study, we thus sought to analyze the contributions of Chinese authors in three major regions of China - the Mainland (ML), Hong Kong (HK), and Taiwan (TW) - to research in the fields of nephrology and urology.

## Methods

This retrospective study examined 61 journals related to nephrology and urology that were selected from the “urology and nephrology systems” category of the Science Citation Index Expanded (SCIE) subjects provided by the Institute for Scientific Information (ISI) [8]. This category included resources for the diagnosis and treatment of genitourinary and kidney diseases: general urology and nephrology publications, and specialized research on the prostate, dialysis and other blood purification techniques, transplantation, and renal failure. *European Urology Supplements*, *Pelvi-Perineologie*, *Revista de Nefrologia Dialisis y Trasplante*, *Urologe*, and *International Urogynecology Journal* were not indexed by Medline and were excluded from this study. Our search of the PubMed database on April 20, 2008 sought articles published in the 61 journals (Appendix S1) between January 1999 and December 2008 by researchers from ML, TW, and HK [9]. The ISSN (print) numbers of the journals (Appendix S1) were used to perform this search. Research output from the three regions of China was identified using the first author's institutional affiliations (ad). Original clinical trials (including cross-sectional and prospective cohort studies), randomized controlled trials (RCT), and case reports were compiled using the publication type categories of the PubMed database.

Three methods were used to compare publication quality. First, the accumulated and average IFs were determined using the ISI's 2007 Journal Citation Reports (JCR) [10]. Second, we quantified citations of articles written by researchers affiliated with Chinese institutions. Third, we identified relevant articles published in the leading general-medicine journals [*The New England Journal of Medicine (NEJM)*, *Journal of the American Medical Association (JAMA)*, *The Lancet*, *British Medical Journal (BMJ)*, and *Annals of Internal Medicine (AIM*)]. Articles related to nephrology and urology were first extracted independently by two reviewers (J Xu and LH Hu), and any disagreement between the reviewers was resolved by viewing the titles, abstracts, and full text if necessary. The number of articles published by each region in the top 10 high-impact nephrology and urology journals was also compared. We determined the 10 most popular nephrology and urology journals containing articles from the three regions of China according to the number of such articles published by each journal.

### Statistical analyses

Statistical analyses were performed using STATA 8.0 software (StataCorp LP, College Station, Texas, US) [11]. Count data are summarized in the Tables and Figures. Trends in the quantity of publication output from the three regions were analyzed using curvilinear regression. The Kruskal-Wallis test was used to detect differences among the three regions, and rank-sum tests were conducted between pairs of regions when necessary. Significance was determined with two-tailed tests and *P* values of less than 0.05 were considered significant.

## Results

### Total number of articles

**Figure 1:**
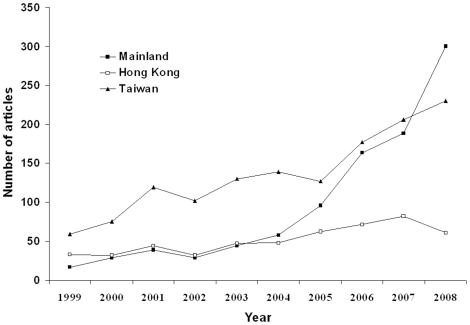
Trends in annual numbers of articles published by researchers from the Mainland (ML), Hong Kong (HK), and Taiwan (TW) from 1999 to 2008.

**Figure 2:**
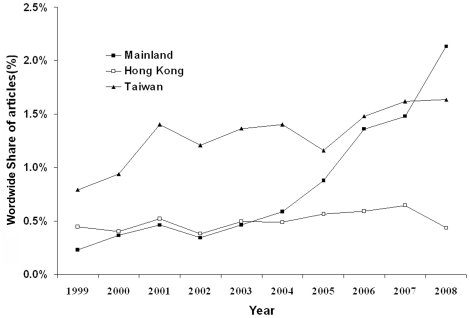
Annual proportion of journal articles in the global literature written by researchers from the Mainland (ML), Taiwan (TW), and Hong Kong (HK) from 1999 to 2008.

A total of 101,632 articles were published in the 61 journals from 1999 to 2008; 2839 (2.8%) of these were from ML (963/2839, 33.9%), HK (512/2839, 18.1%), and TW (1364/2839, 48.0%). The annual number of published articles in the fields of nephrology and urology increased significantly from 1999 to 2008 in the three regions (ML: 17 to 300, r = 0.947, *P*<0.0001; HK: 33 to 61, r = 0.890, *P* = 0.001; TW: 59 to 230, r = 0.929, *P*<0.0001; Fig. 1). The number of articles from ML has exceeded that from HK since 2004, and surpassed that from TW in 2008. The share of articles increased significantly over time in ML (r = 0.899, *P*<0.0001) and TW (r = 0.707, *P* = 0.002), but not in HK (r = 0.270, *P* = 0.12; Fig. 2).

### Clinical trials, randomized controlled trials, and case reports

**Figure 3:**
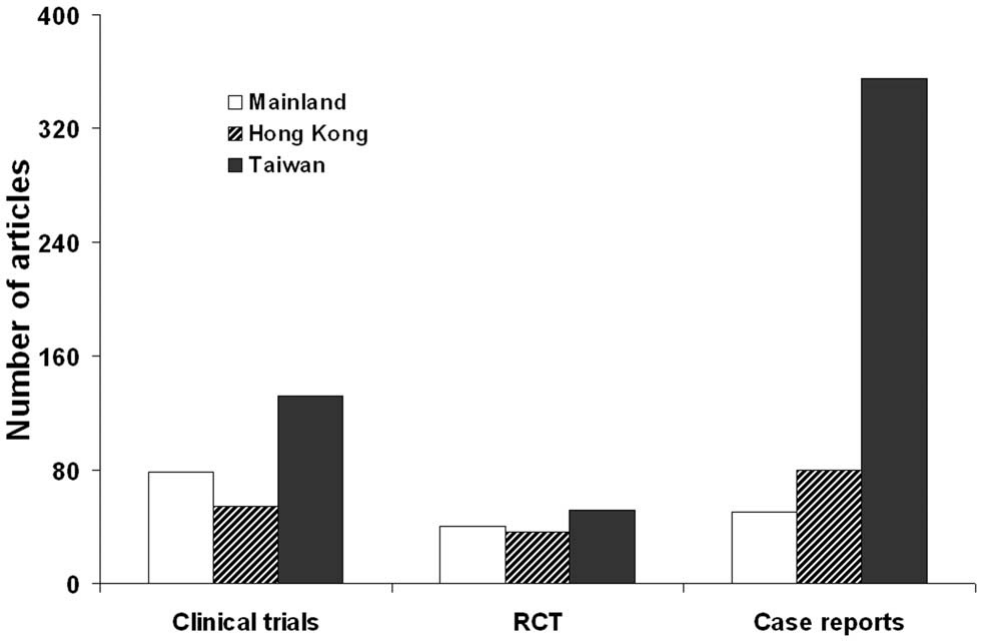
Number of clinical trials, randomized controlled trials (RCT), and case reports published by researchers from the Mainland (ML), Hong Kong (HK), and Taiwan (TW) from 1999 to 2008.

The total quantity of published RCTs did not differ among the three regions (*P* = 0.467). The annual number of published RCTs from ML has exceeded those from HK and TW since 2007, and surpassed the combined total of RCTs from HK and TW in 2008. However, researchers from TW published more clinical trials than those from ML or HK (ML *vs.* TW, *P* = 0.044; HK *vs.* TW, *P* = 0.010). Researchers from TW published 356 case reports between 1999 and 2008, which far exceeded those from ML (*n* = 50, *P*<0.001) and HK (*n* = 80, *P*<0.001). The numbers of case reports from ML and HK did not differ significantly (*P* = 0.863; Fig. 3).

### Impact factors

According to the JCR, 55 nephrology and urology journals had IF in 2007 [10]. Six journals included in our study had no IF. After excluding these journals, we found that the accumulated IF of articles from TW (3620.7) were much higher than those of articles from ML (2272.9) and HK (1589.2, *P* = 0.008). However, articles from HK had the highest average IF (3.2), followed by TW (2.7) and ML (2.3; *P<*0.001; Table 1).

**Table 1:** The accumulated and average impact factors of articles published in nephrology and urology journals by researchers from the Mainland (ML), Hong Kong (HK), and Taiwan (TW) from 1999 to 2008.

Year	Accumulated impact factor			Average impact factor		
	ML	HK	TW	ML	HK	TW
1999	37.0	112.4	177.8	2.2	3.4	3.0
2000	60.0	101.7	220.6	2.1	3.2	2.9
2001	77.9	161.1	341.8	2.0	3.7	2.9
2002	60.3	116.9	266.4	2.1	3.6	2.6
2003	110.5	163.6	305.4	2.5	3.5	2.3
2004	137.3	149.9	346.6	2.4	3.1	2.5
2005	236.7	175.6	332.8	2.5	2.8	2.6
2006	401.5	222.7	455.5	2.5	3.1	2.6
2007	459.7	213.0	566.9	2.4	2.6	2.7
2008	691.9	172.3	606.8	2.3	2.8	2.6
**Total**	**2272.9**	**1589.2**	**3620.7**	**2.3**	**3.2**	**2.7**

### Citations of articles published in nephrology and urology journals

**Figure 4:**
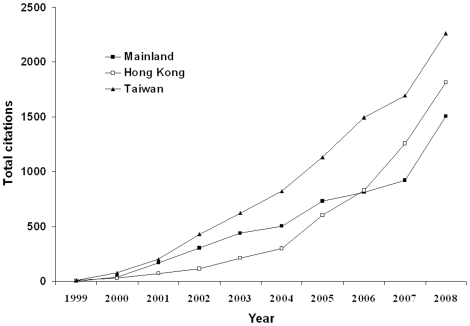
Annual citations of articles published in nephrology and urology journals by researchers from the Mainland (ML), Hong Kong (HK), and Taiwan (TW) from 1999 to 2008.

In this analysis, publications from TW were most frequently cited (8725 citations, 1747 articles), followed by those from HK (5418 citations, 700 articles) and ML (5215 citations, 1680 articles). These differences among the three regions were not significant (*P* = 0.511; Fig. 4).

### High-impact nephrology and urology journals and leading general-medicine journals

A total of 788 articles from the three regions of China were published in the 10 top-ranking nephrology and urology journals. Among them, 17.4% (137/788) were in the top three journals: *Journal of the American Society of Nephrology*, *Journal of Sexual Medicine*, and *European Urology*. Researchers from TW published 398 articles in 10 high-impact nephrology and urology journals, those from HK published 203, and those from ML published 187 articles (Table 2).

**Table 2:** Articles published in the 10 highest-impact nephrology and urology journals by researchers from the Mainland (ML), Hong Kong (HK), and Taiwan (TW) from 1999 to 2008.

Rank	Journal	2007 IF	ML (%)	HK (%)	TW (%)	Total
1	*Journal of the American Society of Nephrology*	7.1	21 (27)	30 (38)	27 (35)	78
2	*Journal of Sexual Medicine*	6.2	5 (38)	1 (8)	7 (54)	13
3	*European Urology*	5.6	20 (43)	2 (4)	24 (52)	46
4	*Kidney International*	4.9	54 (28)	45 (23)	94 (49)	193
5	*Nature Clinical Practice Nephrology*	4.8	1 (10)	8 (80)	1 (10)	10
6	*American Journal of Physiology-Renal Physiology*	4.4	9 (23)	5 (13)	25 (64)	39
7	*Current Opinion in Nephrology and Hypertension*	4.1	1 (50)	1 (50)	0 (0)	2
8	*Journal of Urology*	4.0	45 (24)	16 (9)	126 (67)	187
9	*American Journal of Kidney Diseases*	4.0	14 (8)	81 (46)	82 (46)	177
10	*Prostate*	3.7	17 (40)	14 (33)	12 (28)	43
**Total**			**187 (24)**	**203 (26)**	**398 (51)**	**788**

A total of 263 articles written by Chinese scholars (ML: *n* = 66, TW: *n* = 68, HK: *n* = 129) were published in the five leading general-medicine journals: *NEJM*, *The Lancet*, *JAMA*, *BMJ*, and *AIM*. The two reviewers placed seven of these in the fields of nephrology and urology. HK (RCT: 2, cross-sectional study: 1; *BMJ*: 1, *AIM*: 1, *NEJM*: 1) and TW (RCT: 2, prospective cohort study: 1; *Lancet*: 1, *NEJM*: 1, *AIM*: 1) produced the same number of articles, whereas researchers from ML published only one RCT in *NEJM*.

### Popular nephrology and urology journals

The journals that published the most articles written by Chinese researchers from the three regions are listed in Table 3. For nephrology, *Kidney International* contained the most articles from ML, *American Journal of Kidney Diseases* published the most from TW, and *Nephrology Dialysis Transplantation* contained the most articles from HK. *Kidney International, Nephrology Dialysis Transplantation, Journal of Urology,* and *Nephrology* appeared among the top 10 journals for all three regions.

**Table 3:** The 10 nephrology and urology journals publishing the most articles written by researchers from Mainland China (ML), Hong Kong (HK), and Taiwan (TW).

Rank	ML (*n* = 624)	*N*	HK (*n* = 379)	*N*	TW (*n* = 836)	*N*
1	AJA	233	AJKD	81	*Urology*	158
2	KI	54	PDI	77	NDT	155
3	NDT	52	NDT	60	JU	126
4	*Urology*	50	KI	45	RF	118
5	AJN	48	JASN	30	KI	94
6	BJUI	45	*Nephrology*	26	UI	59
7	JU	45	BJUI	19	*Nephrology*	35
8	*Nephrology*	40	JU	16	PDI	33
9	IJU	31	*Prostate*	14	PN	31
10	JE	26	IJIR	11	JASN	27

AJA, *Asian Journal of Andrology*, IF = 1.6; KI, *Kidney International*, IF = 4.9; NDT, *Nephrology Dialysis Transplantation*, IF = 3.2; AJN, *American Journal of Nephrology*, IF = 2.2; BJUI, *BJU International*, IF = 2.7; JU, *Journal of Urology*, IF = 4.0; IJU, *International Journal of Urology*, IF = 0.8; JE, *Journal of Endourology*, IF = 1.8; AJKD, *American Journal of Kidney Diseases*, IF = 4.0; PDI, *Peritoneal* *Dialysis International*, IF = 2.0; JASN, *Journal of the American Society of Nephrology*, IF = 7.1; IJIR, *International Journal of Impotence Research*, IF = 2.0; RF, *Renal Failure*, IF = 0.6; UI, *Urologia Internationalis*, IF = 2.1; PU, *Pediatric Nephrology*, IF = 1.9; *Urology*, IF = 0.8; *Nephrology*, IF = 1.2; *Prostate*, IF = 3.7.

## Discussion

Nephrology and urology have been practiced in China since the late 1950s, and have developed rapidly since their integration with the international community in the mid-1980s [12]. To our knowledge, this is the first report to assess the contribution of Chinese authors from the major regions of China to nephrological and urological research. The results of the present study clearly indicate that publication by ML researchers in international periodicals increased dramatically during the study period; the number of articles from ML has exceeded that from HK since 2004 and surpassed that from TW in 2008.

As two of the most developed autonomous regions in China, HK and TW have been in the forefront of global scientific research for many years. Researchers in these regions have contributed some of the best nephrological and urological studies to the global literature [13], [14]. Our comparison of publication quality using IF, citation reports, and number of articles published in leading general-medicine journals demonstrated that the gap between ML and the other two regions (HK, TW) remained considerable at the end of the study period (Table 1, Fig. 4). Articles from HK had the highest average IF, followed by those from TW and ML; articles from TW were cited most, those from HK were cited second-most often, and those from ML were cited the least, although these differences were not significant. Researchers from TW and HK also published more papers in high-impact nephrology and urology journals and leading general-medicine journals than those from ML.

A major reason for this disparity among regions is the limited nature of medical resources that must serve a large population across a broad territory in ML, which has restricted the advancement of scientific research. The disparity between urban and vast rural areas has made it difficult for the government to ensure basic health-care services, leaving few resources available for scientific research [15]. In 2002, nearly 1550 pmp in TW received regular dialysis treatment; this prevalence was higher than that in the United States. In the same year, 550 pmp in HK and only 30 pmp in ML received such treatment [16].

Another factor likely affecting the distribution of our results is the use of English as the language of publication for most medical and scientific publications that reach an international audience. Because English has only become widespread in ML since the reform and opening-up policies were initiated in 1978, a substantial amount of high-quality work conducted by ML researchers has been published in Chinese [1].

Although the total numbers of published RCTs did not differ among the three regions, the annual number of such studies from researchers in ML has exceeded those from researchers in TW or HK since 2007, surpassing the combined total of TW and HK in 2008. Clinical research in ML has thus proceeded at a relatively rapid pace, due in part to the compliance of patients and the relatively low cost of the trials conducted. Like many other developing countries, China has experienced dramatic demographic and epidemiological transitions. The major health threats in China's increasingly urbanized and elderly population are chronic diseases, which currently account for more than 75% of all deaths. The prevalence and burden of chronic disease will likely continue to grow in the future. Thus, Chinese researchers should prioritize multicenter prospective studies and RCTs that target the risk factors, prevention, and treatment of kidney and genitourinary diseases [17].

The articles included in this study were retrieved from the computer-generated PubMed search system, which is a comprehensive database of articles published in high-quality medical journals that is administered by the United States' National Center for Biotechnology Information (NCBI) at the National Library of Medicine in Bethesda, Maryland. This study also used data from the JCR, published by the ISI since 1975. This resource is the most comprehensive citation index for scientific literature, covering more than 6000 journals in 2006. Although IF are not an appropriate measure of the scientific quality of individual articles, they are widely accepted as a tool for the comparative evaluation of article sets [18].

Because our study assessed the contribution of Chinese scholars to the international literature, it may address some inherent limitations of research reporting in this literature. First, the journals in our sample were selected from the urology and nephrology category of the SCIE, but some relevant journals were not included in this index. In addition, we limited the author's affiliation to regional terms (China, HK, TW), thus likely omitting articles that only designated particular Chinese cities or provinces. Moreover, our previous study design included the term “Macau,” which was later excluded due to the paucity of affiliated articles. Despite these limitations, we believe that our use of the three regional terms was sufficient to accurately represent nephrological and urological research in the whole of China.

In conclusion, the number of articles published in the fields of nephrology and urology by researchers from the three major regions of China (ML, TW, and HK) all increased significantly from 1999 to 2008. Although the annual number of publications written by ML researchers exceeded those of TW and HK in 2008, the quality of these articles needs to be improved. More prospective studies and RCTs that focus on disease control and prevention should be conducted in ML.

## Supporting Information

Appendix S1 (0.02 MB DOC)
