# Supplementary material for: Scientific Publications in Nephrology and Urology Journals from Chinese Authors in East Asia: A 10-Year Survey of the Literature
Source: PLoS One. 2011 Apr 8;6(4):e14781. doi: 10.1371/journal.pone.0014781 (PMC3072969; doi:10.1371/journal.pone.0014781)
Supplement: Appendix S1 — (0.02 MB DOC) [file pone.0014781.s001.doc]

**Appendix**

The search terms used were: “0210-4806 OR 1548-5595 OR 1368-5538 OR 0001-7868 OR 0272-6386 OR 0250-8095 OR 0363-6127 OR 0003-4401 OR 1008-682X OR 1464-4096 OR 0253-5068 OR 1195-9479 OR 1342-1751 OR 1558-7673 OR 1555-9041 OR 0301-0430 OR 0302-5144 OR 1911-6470 OR 1062-4821 OR 0963-0643 OR 0090-2934 OR 0302-2838 OR 1492-7535 OR 0955-9930 OR 0919-8172 OR 0301-1623 OR 0892-7790 OR 1121-8428 OR 1051-2276 OR 1743-6095 OR 1046-6673 OR 0022-5347 OR 1420-4096 OR 0085-2538 OR 1745-8323 OR 1743-4270 OR 0211-6995 OR 1769-7255 OR 1320-5358 OR 0931-0509 OR 1526-744X OR 1660-2110 OR 1660-2129 OR 1660-2137 OR 0733-2467 OR 0931-041X OR 0896-8608 OR 1166-7087 OR 0270-4137 OR 1365-7852 OR 0886-022X OR 0036-5599 OR 0894-0959 OR 0270-9295 OR 1744-9979 OR 0042-1138 OR 0094-0143 OR 1078-1439 OR 0300-5623 OR 0090-4295 OR 0724-4983” AND “Hong Kong[ad],”, “Taiwan[ad],”, and “China[ad] NOT Hong Kong[ad] NOT Taiwan[ad].”

**Journal list:**

*Adv Chronic Kidney D, Aging Male, Aktuel Urol, Am J Kidney Dis, Am J Nephrol, Am J Physiol-Renal, Ann Urol, Asian J Androl, BJU Int, Blood Purificat, Can J Urol, Clin Exp Nephrol, Clin J Am Soc Nephro, Clin Nephrol, Contrib Nephrol, Curr Opin Nephrol HY, Curr Opin Urol, Dialysis Transplant, Eur Urol, Hemodial Int, Int J Impot Res, Int J Urol, Int Urol Nephrol, J Am Soc Nephrol, J Endoruol, J Nepphrol, J Renal Nutr, J Sex Med, J Urology, Kidney Blood Press R, Kidney Int, Nat Clin Pract Nephr, Nat Clin Pract Urol, Nefrologia, Nephrol Dial Transpl, Nephrology, Nephron Clin Pract, Nephron Exp Nephrol, Nephron Physiol, Neurourol Urodynam, Pediatr Nephrol, Periton Dialysis Int, Prog Urol, Prostate, Prostate Cancer P D, Renal Failure, Scand J Urol Nephrol, Semin Dialysis, Semin Nephrol, Ther Apher Dial, Urol Clin N Am, Urol Int, Urol Oncol-Semin Ori, Urol Res, Urology, World J Urol.*
